# Carer quality of life in Switzerland: a cross-sectional study of parents of children affected by juvenile arthritis

**DOI:** 10.3389/fpubh.2026.1876854

**Published:** 2026-09-10

**Authors:** Matthew Kerry, Michelle Dey, Julia Dratva, Rotraud K. Saurenmann

**Affiliations:** 1 Department of Health, Institute of Public Health, Zürich University of Applied Sciences (ZHAW), Winterthur, Switzerland; 2 Center for Pediatrics, Cantonal Hospital Winterthur, Winterthur, Switzerland

**Keywords:** carer, carer quality of life, health-related quality of life, quality of life, regression

## Abstract

**Background:**

Juvenile idiopathic arthritis (JIA) is a chronic, painful condition that affects 1–2/1000 children in Switzerland. Care-related quality of life (CarerQol) is an important indicator in both public health and health economics studies. The purpose of the study was to examine the proxy associations between CarerQol and various measurement instruments, based on parents’ assessments of their child’s quality of life. These include generic global measures (EuroQol 5-Dimension, EQ-5D), generic domain-specific tools (Patient Reported Outcomes Measurement Information System, PROMIS), which evaluate depression, anxiety, fatigue, and pain interference, and disease-specific measures [health-related QoL (HRQoL) assessed using the Juvenile Arthritis Multidimensional Assessment Report, JAMAR].

**Methods:**

A cross-sectional design using parent surveys and clinician-reported medical information was employed. A sample of 148 parents of JIA-affected children were administered a questionnaire. HRQoL instruments were subjected to the COnsensus-based Standards for the selection of health Measurement INstruments (COSMIN) guidelines for structural validity and internal consistency. Demographic and clinical data, along with HRQoL measures, were analyzed through a series of hierarchical regression analyses focusing on CarerQol.

**Results:**

The measures’ structural validity and internal consistency according to COSMIN criteria were met. In addition to demographic and biographic data, the EQ-5D contributed incremental validity to CarerQol. Conversely, the PROMIS domains failed to add incremental validity, with only depression approaching statistical significance (*p* = 0.06). The disease-specific JAMAR measure added incremental validity (Δ*R*^2^ = 12%); however, this was observed only for the psychosocial HRQoL dimension in forecasting CarerQol.

**Conclusion:**

We found the incremental validity of disease-specific proxy measures over generic and domain-specific instruments in association with CarerQol. Future research may extend these findings by conducting discrete choice experiments to derive tariffs for utility-based scoring of CarerQol within Switzerland. CarerQol was more strongly associated with their disease-specific psychosocial appraisal of their child’s condition than with generic or symptom-based proxy ratings. These findings suggest that addressing parents’ perceptions of the psychosocial impact of JIA may be more relevant for improving CarerQol than focusing solely on the child’s symptoms.

## Background

Juvenile idiopathic arthritis (JIA) is a chronic childhood condition that affects children of all ages. Joint and tendon pain, swelling, and restricted movement, involving the spine, are the primary manifestations of the disease; additionally, JIA may lead to complications in other organs (particularly the eyes), along with general symptoms such as fever, rash, and overall fatigue. Although disease remission can be achieved in the vast majority of cases today, JIA children still require regular doctor visits and ongoing medication to manage their condition control. Additionally, they may experience relapses. The socioeconomic burden of JIA on patients and their families in Switzerland is not yet known.

Care-related quality of life (CarerQol) measures the impact of caregiving by considering both negative aspects (burden, stress, relational problems, and time restrictions) and positive elements (satisfaction and fulfillment) (1). Originally constructed in Netherlands, CarerQol has been translated into a dozen languages (2). Recent meta-analytic evidence positions CarerQol as an important construct related to caregiver happiness and perceived burden (3).

The majority of studies have examined sociodemographic and clinical correlates of CarerQol (3). Recent meta-analytic evidence indicates that, no CarerQol research on CarerQol has been conducted among patient populations with juvenile idiopathic arthritis (JIA) (3), although the international UCAN CAN-Du project has reported on CarerQol data (4, 5). Furthermore, in Switzerland, limited research on CarerQol has been conducted (6), despite recent evidence supporting the substantial costs of JIA, as well as the underrepresented indirect costs in cost analyses (7). Therefore, a scientific gap exists regarding unique predictors of CarerQol.

The current study aimed to examine the unique associations of CarerQol (a proxy HRQoL) in an understudied population of JIA patients. Specifically, we examined the incremental validity of generic, domain-specific, and disease-specific measures in predicting CarerQol, compared to traditional sociodemographic and clinical variables in predicting CarerQol.

## Methods

PAVE is a unique collaboration among international, cross-disciplinary partners in Canada, Germany, Switzerland, Spain, Israel, and Belgium, with projects exploring various topics related to the socioeconomic impacts of JIA, including costs and outcomes, uveitis, mental health, and equity (8). The present study by the Swiss team used a multi-center, cross-sectional observational design with survey methodology.

### Target population

The study targeted children and adolescents aged 0–18 years with a confirmed diagnosis of JIA, as well as their parents. Parents were included as key informants because they provided essential sociodemographic and contextual information. Since only the parents’ responses, along with clinical information, were used in this study, we will provide a detailed explanation of their participation in the following section.

### Study procedure

Participants were recruited through nine pediatric rheumatology outpatient clinics in the German- and Italian-speaking regions of Switzerland. Clinicians informed parents about the study both verbally and in writing. Parents who agreed to participate provided written informed consent, after which clinicians verified eligibility according to predefined inclusion and exclusion criteria.

The inclusion criteria were as follows: (1) a confirmed diagnosis of JIA according to International League Against Rheumatism (ILAR) criteria, (2) child age ≤18 years, (3) parents living with their children at least 3 days per week, (4) access to the Internet to complete the online survey, (5) mental capacity to provide written informed consent, and (6) signed informed consent.

The exclusion criteria included the following: (1) insufficient proficiency in German or Italian language, (2) participation of another child from the same family, (3) participation of more than two parents or legal guardians per child, and (4) missing or revoked informed consent.

Clinicians entered clinical and sociodemographic information about eligible children and their parents into a REDCap database. Personalized links to the online survey were then automatically sent to parents. Reminder emails were sent after 2 and 4 weeks, and clinicians could follow up personally if surveys remained incomplete. The recruitment took place between January 2024 and June 2025. The study protocol (BASEC-Nr. 2023–01069) was approved by the Cantonal Ethics Committee of the Canton of Zurich for all participating hospitals. The research was conducted in accordance with the Declaration of Helsinki.

### Measures

#### Demographics

Parents self-reported demographic information through a questionnaire, which included age, gender, education, and monthly income.

#### Clinical variables

Clinical variables included parent self-reports of the number of current medications, clinician-reported time since diagnosis (years), and clinician-rated disease activity. Disease activity ranged from 0 (inactive) to 10 (highly active).

#### EQ-5D-Y-3L

Generic QoL comprised the five items on the traditional 3-point Likert-type scale from 0 (no problems) to 2 (a lot of problems) (9).

#### Proxy PROMIS domains

Pediatric fatigue, depression, anxiety, and pain interference were assessed by parents for their children using the appropriate forms of the Patient-Reported Outcomes Measurement Information System (PROMIS®). This assessment was administered to parents of children aged ≥5 years. Items per domain varied from 6 to 10, with all items using the same 5-point Likert-type scale response format ranging from 1 (never) to 5 (almost always) (10).

#### Juvenile Arthritis Multidimensional Assessment Report (JAMAR)

The JAMAR Physical Health (*JAMAR*-*PH*) measure comprised five items on a 4-point Likert-type scale, with responses ranging from 0 (no difficulty) to 3 (unable to do). The JAMAR PsychoSocial Health (*JAMAR-PSH*) also comprised five items on a 4-point Likert-type scale, with responses ranging from 0 (never) to 3 (all the time) (11).

#### CarerQol

Care-related Qol was assessed using the seven-item CarerQol-7D (1).

#### Happiness

A single-item measure of carer happiness was assessed on a 10-point scale as an exploratory outcome following the primary analyses.

### Analysis

Data were primarily analyzed using IBM SPSS Statistics version 27.0. The analysis included descriptive statistics, assessments of internal consistency reliability (McDonald’s omega, ω), and hierarchical regressions. Hierarchical regressions ordering generic to more specific measures were conducted, consistent with the World Health Organization international classification system, to postulate the unique contributions of generic and disease-specific measures in health assessments (12, 13). Our primary model was a single-measures prognostic model of CarerQol. A second, exploratory model examined the same associations with carer happiness to examine the nomological net of correlates. That is, we aimed to examine the robustness of proxy predictors for related CarerQol outcomes, such as happiness. Confirmatory factor analyses for model fit indices (structural validity) were conducted using EQS 6.2. The patterns of missing data were examined.

## Results

Of the 148 surveys collected, all demographic and clinical variables were complete.

Among the substantive variables, EQ-5D exhibited no missing data, CarerQol exhibited 2 missing values, JAMAR exhibited 8 missing values, and PROMIS exhibited 11 missing values. One case was missing both JAMAR and PROMIS values. Given the low magnitude of missing data (<14%), we elected listwise deletion for hierarchical regression analyses. It should be noted that this approach necessarily reduces the statistical power for detecting significant predictors in regression models and may introduce bias; however, the latter is unlikely given our results, which indicate missing-completely-at-random missing data mechanisms, *χ*^2^(42) = 47.18, *p* = 0.270. A chi-square test was conducted to compare missingness on our clinical variables, indicating non-significance: *χ*^2^_#meds_ = 5.14, df = 3, *p* = 0.16, *χ*^2^_TimeDiagnosis_ = 7.81, df = 16, *p* = 0.95, *χ*^2^_DiseaseActivity_ = 8.02, df = 9, *p* = 0.53.

The sample had a mean age of 44.7 years (SD = 6.7), with the majority being women (84%). The majority of participants had completed vocational training or tertiary education (82% combined), and 67% reported a monthly household income of CHF 6,000 or higher; children had been diagnosed for an average of 4.18 years (SD = 3.77) and demonstrated low clinician-rated disease activity (M = 1.26, SD = 1.90). Table 1 displays sample characteristics.

**Table 1:** Sample characteristics.

Variable name	Variable label	Value
Sample size (*n*)		148
Age	Years mean (SD)	44.7 (6.7)
Gender: *n* (%)	Women	124 (84)
	Men	24 (16)
Education: *n* (%)	≤Compulsory schooling	7 (4)
	Vocational training	74 (41)
	Vocational/technical baccalaureate	18 (10)
	Tertiary education (uni/applied uni)	73 (41)
	Other	7 (4)
Monthly income*: *n* (%)	<4,500	17 (10)
	4,500–6,000	29 (16)
	6,000–9,000	46 (26)
	>9,000	73 (41)
	Prefer no answer	14 (8)
Child’s number of current medications	Mean (SD)	1.06 (0.81)
Time since diagnosis	Years mean (SD)	4.18 (3.77)
Clinician-rated disease activity Range 0 (inactive)–10 (highly active)	Mean (SD)	1.26 (1.90)
Proxy EQ-5D Range 1 (no problems)–3 (a lot)	Mean (SD)	2.76 (0.30)
PROMIS fatigue Range 1 (never)–5 (almost always)	Mean (SD)	1.70 (0.74)
PROMIS anxiety Range 1 (never)–5 (almost always)	Mean (SD)	1.63 (0.74)
PROMIS depression Range 1 (never)–5 (almost always)	Mean (SD)	1.59 (0.75)
PROMIS pain interference Range 1 (never)–5 (almost always)	Mean (SD)	1.64 (0.85)
JAMAR physical health Range 1 (with no difficulty)–4 (unable to do)	Mean (SD)	2.48 (2.68)
JAMAR psycho-social health Range 0 (never)–3 (all the time)	Mean (SD)	2.49 (2.80)

* Currency values are reported in Swiss francs (CHF), with 1CHF ≈ 1.30 USD. Some variable frequencies vary slightly for 148 participants due to missing data for selected control variables. CarerQol *N* = 146, JAMAR *N* = 140, PROMIS *N* = 137.

Table 2 displays COSMIN-criteria psychometrics for the substantive HRQoL instruments. All instruments demonstrated sufficient structural validity, with confirmatory factor analyses indicating good model fit across measures (CFI ≥ 0.96; SRMR ≤ 0.05). Regarding internal consistency, EQ-5D (*ω* = 0.74), PROMIS domains (*ω* = 0.93–0.95), and JAMAR subscales (*ω* = 0.84–0.90) showed sufficient reliability (*ω* ≥ 0.70). The PROMIS domains demonstrated particularly high reliability. For the CarerQol, the overall total score showed lower consistency (*ω* = 0.57), resulting in an inconsistent rating according to COSMIN thresholds. A reduced version, removing reverse-scored items, exhibited satisfactory reliability (*ω* = 0.79). Both versions were used in analyses to identify potential distortions to results. These findings support the adequate structural validity and reliability of the EQ-5D, PROMIS, and JAMAR measures, whereas the CarerQol total score may require cautious interpretation. It should be noted that additional validity aspects of COSMIN (content, cross-cultural, responsiveness) need further study for examination.

**Table 2:** COSMIN psychometric properties of substantive HRQoL instruments.

COSMIN criteria	EQ-5D	PROMIS (4-Fac: fatigue, anxiety, depression, pain)	JAMAR (2-Fac) physical, psychosocial)	CarerQol
Structural validity	+ CFI = 0.98; SRMR = 0.04	+ CFI = 0.97; SRMR = 0.03	+ CFI = 0.96; SRMR = 0.05	+ CFI = 0.97; SRMR = 0.03
Internal consistency	+ *ω* = 0.74	+ *ω* = 0.95, 0.94, 0.93, 0.94	+ *ω* = 0.90, 0.84	± *ω* = 0.79; *ω*_(total)_ = 0.57

*N* = 148. COSMIN ratings: +, sufficient; −, insufficient; ±, inconsistent; ?, indeterminate. Fat, fatigue; Dep, depression; Anx, anxiety; PH, physical health; PSH, psychosocial health. ω, McDonald’s omega reliability coefficient. Fit indices: CFI, comparative fit index; TLI, Tucker–Lewis index; SRMR, standardized root mean square residual.

Table 3 presents hierarchical regression results for CarerQol. In Model 1, sociodemographic and clinical variables explained 15.1% of the variance in CarerQol (*R*^2^ = 0.151, *F*(7,121) = 3.08, *p* = 0.005). A higher number of medications was significantly associated with poorer CarerQol (*β* = −0.23, *p* = 0.009). No other variables were statistically significant. In Model 2, the addition of generic global EQ-5D significantly improved model fit, explaining an additional 9.7% of the variance (Δ*R*^2^ = 0.097, *F* change = 15.46, *p* < 0.001; total *R*^2^ = 0.248). Better EQ-5D scores were associated with higher CarerQol (*β* = 0.34, *p* < 0.001). The effect of number of medications remained significant (*β* = −0.18, *p* = 0.028).

**Table 3:** Hierarchical linear regressions of CarerQol.

Variables	M1 B (SE)	*β*	*p*	M2 B (SE)	*β*	*p*	M3 B (SE)	*β*	*p*	M4 B (SE)	*β*	*p*
Age	0.24 (0.25)	0.09	0.326	0.23 (0.23)	0.09	0.328	0.26 (0.23)	0.10	0.253	0.03 (0.21)	0.01	0.886
Gender	−0.49 (0.36)	−0.12	0.174	−0.47 (0.34)	−0.11	0.168	−0.37 (0.33)	−0.09	0.271	−0.44 (0.30)	−0.10	0.154
Education	0.24 (0.13)	0.17	0.057	0.21 (0.12)	0.15	0.072	0.12 (0.12)	0.09	0.310	0.12 (0.11)	0.08	0.303
Income	0.01 (0.01)	0.12	0.190	0.01 (0.01)	0.11	0.197	0.01 (0.01)	0.09	0.285	0.01 (0.01)	0.07	0.365
Medications	**−0.46 (0.17)**	−0.23	**0.009**	**−0.37 (0.17)**	−0.18	**0.028**	−0.17 (0.17)	−0.09	0.313	−0.06 (0.16)	−0.03	0.728
Time since dx	−0.01 (0.04)	−0.03	0.757	−0.02 (0.03)	−0.04	0.654	−0.02 (0.03)	−0.04	0.633	0.01 (0.03)	0.03	0.690
Disease activity	−0.10 (0.07)	−0.13	0.153	−0.00 (0.07)	−0.00	0.975	−0.00 (0.07)	−0.00	0.970	0.00 (0.07)	0.00	0.973
EQ-5D	—	—	—	**0.37 (0.09)**	0.34	**<0.001**	0.15 (0.12)	0.14	0.193	0.11 (0.12)	0.10	0.341
Fatigue	—	—	—	—	—	—	−0.02 (0.03)	−0.11	0.399	0.00 (0.03)	0.01	0.933
Anxiety	—	—	—	—	—	—	0.02 (0.04)	0.06	0.643	0.03 (0.03)	0.09	0.470
Depression	—	—	—	—	—	—	−0.10 (0.05)	−0.26	0.063	−0.02 (0.05)	−0.06	0.636
Pain interference	—	—	—	—	—	—	−0.03 (0.03)	−0.11	0.406	−0.03 (0.03)	−0.14	0.285
JAMAR Physical	—	—	—	—	—	—	—	—	—	0.06 (0.08)	0.10	0.483
JAMAR Psychosocial	—	—	—	—	—	—	—	—	—	**−0.35 (0.08)**	−0.54	**<0.001**
Model *R*^2^	0.151			0.248			0.324			0.439		
Δ*R*^2^	—			0.097			0.076			0.116		
F change (*p*)	**3.076 (0.005)**			**15.460 (<0.001)**			**3.252 (0.014)**			**11.750 (<0.001)**		

*N* = 128 due to missing control variable values. All VIFs < 5 and all tolerances > 0.20. Bold values = *p* < 0.05.

In Model 3, PROMIS domains (fatigue, anxiety, depression, and pain interference) were added and accounted for a further 7.6% of variance (Δ*R*^2^ = 0.076, *p* = 0.014; total *R*^2^ = 0.324). None of the PROMIS domains reached statistical significance, although depressive symptoms showed a trend toward poorer CarerQol (*β* = −0.26, *p* = 0.063). Previously significant variables were no longer significant. Finally, in Model 4, JAMAR HRQoL domains were included, producing the largest improvement in explanatory power (Δ*R*^2^ = 0.116, *p* < 0.001), with the final model explaining 43.9% of the variance (*R*^2^ = 0.439, *F*(14,114) = 11.75, *p* < 0.001). Psychosocial HRQoL was strongly associated with CarerQol (*β* = 0.54, *p* < 0.001), whereas physical HRQoL and all other variables were not statistically significant. It should also be noted that, in the sensitivity analysis in which all items of the CarerQol measure were included, no differences were observed in the final model with respect to variable significance. In particular, the greatest parameter departure was Δ*β = 0*.02. The psychosocial factor of the JAMAR remained the only significant predictor, with the coefficient increasing slightly from *β* = −0.35 to *β* = −0.48.

A final, exploratory analysis aimed to examine the robustness of JAMAR’s incremental validity on focal outcomes by repeating the hierarchical regressions on a 10-point, single-item indicator of carer happiness. The results were largely replicated, but extended further. In the final-adjusted model, EQ-5D remained significant, PROMIS-Pain Interference was significant, and both JAMAR subscales (physical and psychosocial health) were significant correlates of carer happiness (see Table 4).

**Table 4:** Hierarchical linear regressions of carer happiness.

Variables	M1 B (SE)	*β*	*p*	M2 B (SE)	*β*	*p*	M3 B (SE)	*β*	*p*	M4 B (SE)	*β*	*p*
Age	−0.11 (0.30)	−0.03	0.731	−0.13 (0.28)	−0.04	0.651	−0.10 (0.27)	−0.03	0.694	−0.15 (0.27)	−0.05	0.583
Gender	−0.42 (0.44)	−0.08	0.340	−0.39 (0.40)	−0.08	0.334	−0.29 (0.39)	−0.06	0.455	−0.37 (0.38)	−0.07	0.334
Education	0.21 (0.15)	0.12	0.173	0.17 (0.14)	0.10	0.226	0.06 (0.14)	0.03	0.693	0.10 (0.14)	0.05	0.490
Income	0.02 (0.01)	0.15	0.093	0.02 (0.01)	0.14	0.087	0.01 (0.01)	0.11	0.172	0.01 (0.01)	0.09	0.248
Medications	−0.40 (0.21)	−0.17	0.062	−0.26 (0.20)	−0.11	0.187	0.00 (0.20)	0.00	0.999	0.07 (0.20)	0.03	0.735
Time since dx	0.03 (0.04)	0.07	0.478	0.03 (0.04)	0.05	0.532	0.03 (0.04)	0.06	0.480	0.04 (0.04)	0.08	0.317
Disease activity	−0.13 (0.09)	−0.13	0.150	0.02 (0.08)	0.03	0.782	0.02 (0.09)	0.02	0.840	−0.04 (0.09)	−0.04	0.628
EQ-5D	—	—	—	**0.55 (0.11)**	0.43	**<0.001**	**0.28 (0.14)**	0.21	**0.045**	**0.39 (0.14)**	0.30	**0.008**
Fatigue	—	—	—	—	—	—	0.00 (0.03)	0.02	0.897	0.01 (0.03)	0.03	0.814
Anxiety	—	—	—	—	—	—	0.01 (0.04)	0.03	0.823	0.03 (0.04)	0.10	0.436
Depression	—	—	—	—	—	—	**−0.13 (0.06)**	−0.30	**0.028**	−0.10 (0.06)	−0.22	0.111
Pain interference	—	—	—	—	—	—	−0.06 (0.04)	−0.20	0.123	**−0.10 (0.04)**	−0.35	**0.014**
JAMAR physical	—	—	—	—	—	—	—	—	—	**0.27 (0.10)**	0.40	**0.009**
JAMAR psychosocial	—	—	—	—	—	—	—	—	—	**−0.24 (0.09)**	−0.31	**0.013**
Model *R*^2^	0.120			0.270			0.363			0.411		
Δ*R*^2^	—			0.150			0.093			0.048		
*F* change (*p*)	**2.349 (0.028)**			**24.705 (<0.001)**			**4.236 (0.003)**			**4.675 (0.011)**		

*N* = 128 due to missing control variable values. All VIFs < 5 and all tolerances > 0.20. Bold values = *p* < 0.05.

## Discussion

This study provides new evidence regarding the psychometric performance and convergent validity of CarerQol in a Swiss population using a cross-sectional survey design.

CarerQol was more strongly associated with parents’ disease-specific psychosocial appraisal of their child’s condition than with generic or symptom-based proxy ratings. These findings suggest that addressing parents’ perceptions of the psychosocial impact of JIA on their child may be more relevant for improving CarerQol than focusing on the child’s symptoms alone.

Although we identified psychosocial factors as unique correlates of CarerQol, it should be noted that our final model only accounted for approximately 44% of variance, suggesting more predictor space to be identified in future research. The extension of our findings to carer happiness also aligns with recent meta-analytic findings (3). It appears, however, that carer happiness is associated with both physical and psychosocial factors related to the child’s arthritic condition. These findings should have implications for both the clinical support systems available to informal caregivers and public health interventions aimed at preventing caregiver burnout (14).

The cross-sectional design of our study prevents conclusions about causal pathways. It remains unclear whether parents with lower CarerQol appraise the child’s psychosocial difficulties as more severe or whether the child’s psychosocial burden drives caregiver strain. Longitudinal research is needed to disentangle these processes.

### Limitations

First, internal consistency was insufficient or borderline, which is consistent with recent meta-analytic findings (3). Although lower reliability is a well-known cause of regression model insignificance, it should be noted that we conducted a sensitivity analysis and found no substantive differences (changes in significance) between the full and reduced measures of CarerQol. However, future research should assess whether the full instrument demonstrates adequate reliability (*ω* > 0.70) for substantive analyses. It should also be noted that the full measure may be desirable for retention toward identifying potential careless responders. The identification of the two reverse-scored items being problematic may indicate careless responding issues. Future studies with CarerQol as a focal variable may improve careless responding using screening items and administering the instrument early in questionnaires (15).

Second, our sample was limited to parents of juvenile idiopathic arthritis patients in the German- and Italian-speaking parts of Switzerland. The generalizability of our findings should be examined in the French-speaking part of Switzerland. Slightly larger sample sizes (*n* > 200) may also permit item bias testing across Swiss languages (16). Relatedly, our findings of incremental value of disease-specific measures over generics for CarerQol should be examined in new disease populations. Relatedly, given our relatively low disease activity in our current sample, the generalizability of our findings should be examined in active-JIA patients.

Third, one of the primary goals of CarerQol is to develop standardized quality-of-life scores for use in economic studies to understand the impact of caregiving (2). Although beyond the purview of our study, our preliminary psychometric validation should encourage future discrete choice experiments in Switzerland to further CarerQol’s practical application in health-economic studies.

Fourth, this cross-sectional study exhibited sufficient construct validity and internal consistency evidence for the CarerQol. However, this “static” evidence represents only one aspect of measurement quality. Repeated-measures studies are required to further examine CarerQol responsiveness and measurement error—both of which are critical to understanding CarerQol’s appropriateness for monitoring changes over time and assessing intervention effects.

Fifth, we should also note the necessary common method bias inherent to the same-source measures (parent reports) throughout our study (17). For example, a meta-analysis on common method bias in health research reported that the effect can vary widely between constructs, ranging from no effect to approximately 64% inflation (18). Several studies emphasize the substantive differences between parent- and other-reports (19, 20). Although not a focus of our current study, a follow-up study examines directional discrepancies between parent and child reports of Qol measures (21).

## Conclusion

The included HRQoL instruments demonstrated overall adequate measurement properties, except for internal consistency. In hierarchical regressions, generic proxy instruments added incremental validity over traditional sociodemographic and clinical variables for CarerQol. Physical health indicators and PROMIS symptom domains did not contribute independently in the fully adjusted model, whereas the disease-specific JAMAR psychosocial subscale emerged as the strongest predictor of CarerQol. These findings highlight the importance of proxy psychosocial measures in understanding caregiver outcomes and support the use of psychometrically robust HRQoL measures in caregiver research and intervention planning.

Our findings suggest that routine clinical care in pediatric rheumatology should incorporate systematic screening of caregiver burden and psychosocial wellbeing. Interventions targeting illness perception, coping strategies, and family functioning (e.g., psychoeducation and cognitive-behavioral support) may be particularly relevant to improve CarerQol.

## Data Availability

The raw data supporting the conclusions of this article will be made available by the authors, without undue reservation.

## Group member of PAVE consortium

Canada: Deborah A. Marshall, Brittany Gerber, Gillian R. Currie, Susanne M. Benseler, Tania Cellucci, Guillermo Fiebelkorn, Sara A. Kohut, Jennifer N. Stinson, Elizabeth Stringer, Joost F. Swart, Stacey A. Page, Nico Wulffraat, and Rae S. M. Yeung. Germany: Kirsten Minden, Jens Klotsche, Juliane Riess, Florian Milatz, Martina Niewerth, Claudia Sengler, Nadine Grösch, and Ina Liedmann. Switzerland: Rotraud K. Saurenmann, Michelle Dey, Sibylle Juvalta, Pascale Kurt, and Julia Dratva. Eva Adank, Florence Aeschlimann, Elvira Cannizzaro, Christian Huemer, Daniela Kaiser, Klaus Tenbrock, Federica Vanoni, Andreas Wörner. Sonja Feer, Daniela Herdlicko, Matthew Kerry, Felix Moerman, Seraina Palmer-Sarott, Lisa Pedrotti, Johannes Roth, Silke Schroeder, David Shaw, Christa Soennichsen, and Tatjana Welzel. Spain: Jordi Antón, Laura Martinez Mifsut, Mercè Jabalera, Joan Vinyets, Elisabet Puiggros Ruiz, Sonia Carriquí Arenas, Nolvia Castillo Giron, Joan Calzada Hernandez, Estibaliz Iglesias Jiménez, Juan M. Mosquera, Andrea Montserrat Zacarias Crovato, Carolina Estepa Guillen, Patricia Ramírez Rodríguez, Marina Barraso Rodrigo, Jesús Díaz Cascajosa, Ana Isabel Díaz Cortés, Marta Morales Ballus, Marta Llorens Capdevila, Maria Crespo Bosch, Ariadna Riera Castello, Montserrat Salvado Gavalda, Marta Gonzalez Martinez, and Alexia Reyes Segura. Israel: Yosef Uziel, Amit Ziv, Lia Henan, Ruby Haviv, Shirley Alon, Merav Heshin, Irit Tirosh, Yonathon Butbul Aviel, Neta Aviran, Rotem Semo, Mohamad Hamad, Gil Amarylio, and Chen Dagan. Belgium: Carine Wouters, Lien De Somer, Lien Lijnen, Joanna Willen, Kristien Hens, Leni Van Goidsenhoven, and Hilde De Tollenaere. Patient Advocacy Organizations: Jennifer Wilson, David Porte, Tsipi Egert, Yona Egert, and Christophe Normand.
